# Estrogen receptor-α is required for the osteogenic response to mechanical loading in a ligand-independent manner involving its activation function 1 but not 2

**DOI:** 10.1002/jbmr.1754

**Published:** 2013-02

**Authors:** Sara H Windahl, Leanne Saxon, Anna E Börjesson, Marie K Lagerquist, Baruch Frenkel, Petra Henning, Ulf H Lerner, Gabriel L Galea, Lee B Meakin, Cecilia Engdahl, Klara Sjögren, Maria C Antal, Andrée Krust, Pierre Chambon, Lance E Lanyon, Joanna S Price, Claes Ohlsson

**Affiliations:** 1 Department of Medicine and Clinical Nutrition, Centre for Bone and Arthritis Research, Institute of Medicine, Sahlgrenska Academy, University of GothenburgGothenburg, Sweden; 2 The Royal Veterinary CollegeLondon, UK; 3 Department of Orthopaedic Surgery, Keck School of Medicine, University of Southern CaliforniaLos Angeles, CA, USA; 4 Department of Biochemistry and Molecular Biology, Keck School of Medicine, University of Southern CaliforniaLos Angeles, CA, USA; 5 Department of Molecular Peridontology, Molecular Periodontology, Umeå UniversityUmeå, Sweden; 6 School of Veterinary SciencesBristol, UK; 7 Institut de Génétique et de Biologie Moléculaire et Cellulaire (CNRS, INSERM, UdS, Collège de France)Illkirch, Strasbourg, France

**Keywords:** ESTROGEN, RECEPTOR, MECHANICAL, LOADING, MOUSE, BONE

## Abstract

Estrogen receptor-α (ERα) is crucial for the adaptive response of bone to loading but the role of endogenous estradiol (E2) for this response is unclear. To determine in vivo the ligand dependency and relative roles of different ERα domains for the osteogenic response to mechanical loading, gene-targeted mouse models with (1) a complete ERα inactivation (ERα^−/−^), (2) specific inactivation of activation function 1 (AF-1) in ERα (ERαAF-1^0^), or (3) specific inactivation of ERαAF-2 (ERαAF-2^0^) were subjected to axial loading of tibia, in the presence or absence (ovariectomy [ovx]) of endogenous E2. Loading increased the cortical bone area in the tibia mainly as a result of an increased periosteal bone formation rate (BFR) and this osteogenic response was similar in gonadal intact and ovx mice, demonstrating that E2 (ligand) is not required for this response. Female ERα^−/−^ mice displayed a severely reduced osteogenic response to loading with changes in cortical area (−78% ± 15%, *p* < 0.01) and periosteal BFR (−81% ± 9%, *p* < 0.01) being significantly lower than in wild-type (WT) mice. ERαAF-1^0^ mice also displayed a reduced response to mechanical loading compared with WT mice (cortical area −40% ± 11%, *p* < 0.05 and periosteal BFR −41% ± 8%, *p* < 0.01), whereas the periosteal osteogenic response to loading was unaffected in ERαAF-2^0^ mice. Mechanical loading of transgenic estrogen response element (ERE)-luciferase reporter mice did not increase luciferase expression in cortical bone, suggesting that the loading response does not involve classical genomic ERE-mediated pathways. In conclusion, ERα is required for the osteogenic response to mechanical loading in a ligand-independent manner involving AF-1 but not AF-2. © 2013 American Society for Bone and Mineral Research

## Introduction

Cortical bone dimensions have been reported to be the main determinant of bone strength and it is well established that mechanical loading and estrogen receptor (ER)-mediated pathways are major regulators of cortical bone dimensions.^1–3^ Bones are believed to have a strain-driven feedback system that senses the incident mechanical strain within the loaded bones. Subsequently, bone tissue is removed from sites where the loading is marginal and new bone is formed at sites subjected to increased loading in order to provide each bone with a mechanically appropriate size, shape, and architecture.^4^, ^5^

Estrogens are known to protect against bone loss and this is primarily mediated by ERα.^6–11^ The possible role of ERα for the osteogenic response to loading has been evaluated in female mice with a compromised ERα expression. These mice display a significantly reduced anabolic response on cortical bone area to mechanical loading.^12–15^ The ERα knockout mouse model used in these experiments, developed in the Korach and Smithies laboratories (National Institute of Health, NC, USA) (K-ERα^−/−^), was shown to have a low expression of truncated ERα isoforms, possibly compromising the interpretation of the results.^16^ In addition, a role for ERα in humans is supported by an association study suggesting that genetic variants at the *ERα* locus modulate the mechanosensitivity of bone.^17^ These findings support the hypothesis that ERα number and/or function in bone cells may limit the bones' adaptability to mechanical loading. In contrast, the in vivo data concerning the role of ERβ for the osteogenic response to loading is conflicting, reflected by the fact that mice deficient in ERβ (ERβ^−/−^) either display a reduced^13^ or enhanced^18^ osteogenic response to loading.

ERα stimulates gene transcription via two activation functions (AFs), AF-1 in the N-terminal and AF-2 in the ligand binding domain. We have recently reported that the effect of estradiol (E2) on cortical bone in ovariectomized (ovx) mice is dependent on AF-2 but not AF-1 in ERα.^1^ However, the relative roles in vivo of ERαAF-1 and ERαAF-2 for the ERα-mediated effects of mechanical loading in cortical bone are unknown.

The classical activation of genes via ERα includes hormone-receptor binding followed by activation of genes with estrogen response element (ERE)-containing promoters. Both mechanical strain and E2 increase the transcriptional activity from an ERE-reporter transiently transfected into an osteoblast cell-line, indicating that both strain and E2 enhance osteoblast activity via ERE-mediated mechanisms in vitro. However, it is not yet determined in vivo if ERE-mediated mechanisms are involved in the osteogenic response to mechanical loading.^19^

Although it is clear that ERα is required for a normal osteogenic response to loading, contradictory data exist concerning the role of E2 for this response. Estrogen has been shown to increase,^20^ decrease,^4^ or not affect^21^, ^22^ the osteogenic response to exercise. Using male rats, it was shown that low-dose E2 treatment suppresses cortical periosteal bone formation in response to axial mechanical loading of the ulna.^23^ In contrast, no effect of ovx was seen on the cortical bone response to external loading of tibia by a four-point bending device^24^ or unloading of the left hind limb in female rats.^25^ Thus, it is still unclear whether estrogen is involved in the osteogenic effect of loading.

To determine in vivo the ligand (E2) dependency and the relative roles of different ERα domains for the osteogenic response to mechanical loading, gene-targeted female mouse models with (1) a complete ERα inactivation (ERα^−/−^), (2) specific inactivation of AF-1 in ERα (ERαAF-1^0^), or (3) specific inactivation of ERαAF-2 (ERαAF-2^0^) were subjected to short periods of cyclic compressive loading of the tibia, three times a week for 2 weeks, in the presence (sham) or absence (ovx) of E2.

## Subjects and Methods

### Animals

The mice were inbred on a C57BL/6 background and housed in a standard animal facility under controlled temperature (22°C) and photoperiod (12 hours of light, 12 hours of dark), and fed *ad libitum*. Littermate controls were used in all groups. All animal experiments were approved by the local Ethical Committee for Animal Research. The generation of ERα^−/−^,^26^ ERαAF-1^0^,^27^ ERαAF-2^0^,^1^ and transgenic 3xERE-TAT-Luc (ERE-luciferase^28^) mice have been described. In the ovx experiments, the mice were either sham-operated or ovariectomized under inhalation anesthesia with isoflurane (Forene; Abbot Scandinavia, Solna, Sweden) 5 days prior to loading. The effectiveness of ovx was confirmed by measuring the uterine wet weight (WT sham 77.4 ± 11.6 mg, WT ovx 14.0 ± 0.4 mg, ERα^−/−^ sham 12.6 ± 1.9 mg, ERα^−/−^ ovx 7.2 ± 1.1 mg).

### Mechanical strain measurement during dynamic axial loading of the tibia

The magnitude of axial mechanical strain applied to the tibia during loading was established ex vivo in the different evaluated mouse strains. A single-element strain gauge (EA-06-015DJ-120; Vishay Measurement Group, PA; Load Indicator System AB, Gothenburg, Sweden) was bonded with cyanoacrylate adhesive in longitudinal alignment to the medial aspect of the tibia at 37% of its length from the proximal end. Previous studies have shown that this region corresponds to the site of greatest osteogenic response to axial loading.^29^ Strains were measured across a range of peak compressive loads between 6 and 14 N. These peak loads were applied with a ramped trapezoidal waveform using a servohydraulic machine (Dartec HC10; Zwick Roell, Herefordshire, UK) with the same holding cups that were used for in vivo loading. When the axial force is applied to the tibia, the bone bends in the medial-lateral direction resulting in tension on the medial surface and compression on the lateral surface.^30^ From the data, a specific peak load (in N) corresponding to 3050 ± 120 µ at the gauge site was used for each group of mice in the loading experiment (load in N, ERαAF-1^0^ 12 N; WT [siblings to ERαAF-1^0^] 12 N; ERαAF-2^0^ 11 N; WT [siblings to ERαAF-2^0^] 11 N; ERα^−/−^ mice 10.5 N; WT [siblings to ERα^−/−^] 12 N; ovx ERα^−/−^ 10.5 N; ovx WT [siblings to ERα^−/−^] 12 N). This was selected to engender an osteogenic response without causing damage to the bones, joints, or the skin through which the load was applied.

### In vivo loading of the tibia

While under inhalation anesthesia with isoflurane (Forene), the right tibia of 17-week-old female ERα^−/−^, ERαAF-1^0^, ERαAF-2^0^ mice, and their wild-type (WT) littermates was axially loaded on 3 alternate days per week for 2 weeks for 40 cycles/day with a trapezoid waveform, with 10 seconds of rest between cycles. The loads were applied using a 3100 ElectroForce Test Instrument (Bose Corporation, MN). The left tibia was used as a non-loaded control to allow side-to-side comparisons for the effects of loading on bone (re)modeling. The use of the contralateral limb as a control using this protocol has been validated in our laboratory by comparing remodeling in the bones of limbs contralateral to those used in loading experiments with that in normal limbs of separate animals to which no loads had been applied.^31^ All mice were allowed normal cage activity in between loading sessions. At 19 weeks of age, the mice were euthanized and their tibias dissected free of soft tissue, fixed for 48 hours in Bürkhardt's solution, and stored in 70% ethanol. The ERE-luciferase mice (12 weeks old) were loaded once (40 cycles), 3 or 8 hours before euthanasia.

### Micro–computed tomography

Cortical micro–computed tomography (µCT) analyses were performed on the mid-diaphyseal part of the tibia by using a Skyscan 1072 scanner (Skyscan N.V., Aartselaar, Belgium), imaged with an X-ray tube voltage of 100 kV and current 98 µA, with a 1-mm aluminum filter.^32^ The scanning angular rotation was 180 degrees and the angular increment was 0.9 degrees. The voxel size was 6.51 µm isotropically. Datasets were reconstructed using a modified Feldkamp algorithm^33^ and segmented into binary images using adaptive local thresholding.^34^

### Histomorphometric analyses

Bone formation rate (BFR) at the periosteal and endosteal surfaces of the cortical bone in the mid-diaphyseal region of tibia were evaluated by using dynamic histomorphometric analyses. Tibiae were fixed in Bürckhardt's fixative, dehydrated in increasing concentrations of EtOH, and embedded in plastic (L R White Resin; Agar Scientific, Stansted, UK). For the measurement of dynamic parameters, the mice were double-labeled with calcein and alizarin, which were injected (intraperitoneally [i.p.]) into the mice at the first day (30 mg/kg/d of calcein) and last day (30 mg/kg/d of alizarin) of loading. Histomorphometric analyses of cortical bone were performed using transverse cross-sections in the mid-diaphyseal region of the tibiae. The parameters were measured using the OsteoMeasure histomorphometry analysis system with software version 2.2 (OsteoMetrics Inc, Decatur, GA, USA), and following the guidelines of the American Society for Bone and Mineral Research.^35^

### Peripheral quantitative computed tomography

Peripheral quantitative computed tomography (pQCT) scans were performed with the PQCT XCT RESEARCH M (version 4.5B; Norland, Fort Atkinson, WI, USA), operating at a resolution of 70 µm as described.^10^ Cortical bone parameters (cortical bone mineral content, cortical bone area, polar moment of inertia, and polar moment of resistance) were analyzed ex vivo in the mid-diaphyseal region of tibia.^36^

### Protein preparation and luciferase analysis

Cortical diaphyseal bone from the tibia was homogenized in lysis buffer (25 mM Tris pH 7.8, 1.5 mM EDTA, 10% glycerol, 1% Triton X-100, 2 mM dithiothreitol [DTT] and complete protease inhibitors; Roche #1169749800 Roche Diagnistics, Mannhein, Germany) and separated by centrifugation at 10,650*g* for 30 minutes. The supernatant was stored at −20°C until further analysis. Protein from cell fractions was prepared using Reporter Lysis buffer from the Luciferase Assay (#E4550; Promega, Madison, WI, USA) according to the manufacturer's instructions. The protein content was measured using BioRad DC protein assay (#500-0116). The luciferase activity measurements were performed using a standard Luciferase Assay (#E4030; Promega) according to the manufacturer's instructions and measured on a luminometer (Turner Designs TD-20/20; Promega).

### Cell culture and in vitro loading of osteoblasts

Osteoblasts were cultured from explants of cortical bone of femurs and tibiae of 6-month-old female mice as described.^37^ Briefly, attendant soft tissue was removed from the bones and bone marrow was flushed out with PBS. The bones were cut into approximately 1-mm^2^ fragments and cultured in α modified essential medium (α-MEM) (Gibco, Invitrogen, Auckland, New Zealand) supplemented with 10% fetal bovine serum (FBS) (Sigma–Aldrich, Stockholm, Sweden), 2 mM glutamax (Gibco), 50 µg/mL gentamicin (Gibco), and 100 U/mL penicillin–100 µg/mL streptomycin (PEST; Gibco) for 1 week. Bone fragments were then removed, media changed, and 4 days thereafter the cells were passaged and used for in vitro loading. First passage osteoblasts were cultured on custom-made plastic slides (250,000 cells/slide) and subjected to a single brief period of 600 cycles of four-point bending at a frequency of 1 Hz as described.^38^, ^39^ The waveform of each strain cycle consisted of a ramped square wave with strain rates on and off of 23,000 µ/s, dwell times on and off of 0.4 and 0.75 seconds, respectively, and a peak strain of 3400 µ. Following strain treatment, the cells were maintained in the same media and cultured for 1, 3, or 8 hours (*n* = 10–16 per time point). Static control cells were maintained similarly but not subjected to the strain stimulus. To compare the expression of *Sost* and *DMP1* in these cells with the expression of those transcripts in long bone, RNA was extracted from flushed control murine tibiae as described by our group,^39^, ^40^ and converted to cDNA as described for the in vitro studies below (with reverse transcriptase [RT] as a positive control or lacking RT as a negative control). Using the culture conditions required for the in vitro loading procedure, these cells do not express the osteocyte marker *Sost*. However, these cells do express *DMP1*, a marker of cells that are highly differentiated along the osteoblastic lineage (Supplemental Fig. S1A). *DMP1* expression was not different between WT and ERαAF1^0^ cells (Supplemental Fig. S1B) and the expression of this gene was not significantly influenced by strain in either genotype at the time points tested (Supplemental Fig. S1C).

### RNA preparation and analyses of *Cox-2*, *Egr2*, and *IL-11* mRNA levels

Total RNA was extracted and genomic DNA removed from static and strained cells as previously described using RNEasy Plus kits (Qiagen, Sussex, UK) according to the manufacturer's instructions.^38^, ^39^, ^41^ RNA quality and quantity was determined using a NanoDrop ND1000 (Thermo, UK) and 2 µg of RNA was converted to cDNA using Superscript II (Invitrogen, Paisley, UK). Quantitative RT PCR (qRT-PCR) was then performed as described.^38^, ^39^, ^41^ A relative standard curve was constructed for each gene using serial dilutions of their amplicons, and these standards were included in each run. Samples of unknown concentrations were quantified relative to these standard curves. The expression levels for all the genes analyzed were normalized to the reference gene *β2-MG*. The primer sequences for *Egr2* were as described.^39^ Those for *Ptgs2* (*Cox-2*) and *β2-MG* were as follows: *Cox-2* forward GCTCAGTTGAACGCCTTTTGA and reverse CACAGCCTACCAAAACAGCCA, *β2-MG* forward ATGGCTCGCTCGGTGACCCT and reverse TTCTCCGGTGGGTGGCGTGA. The IL-11 primers were as described.^42^ The Sost primers were as follows: forward TGCCGCGAGCTGCACTACAC and reverse CCCACTTCACGCGCCCGAT. The DMP1-primers were as follows: forward CACCACCACCACCCACGAACA and reverse GGCCTCTGTCGTAGCCCAGC.

## Results

### Endogenous estradiol is not required for the cortical osteogenic response to mechanical loading in female mice

**Figure 1:**
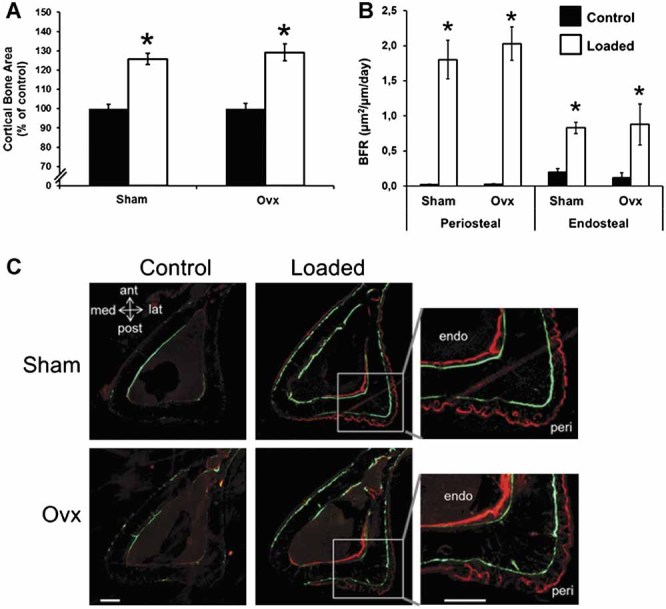
Endogenous estradiol is not required for the cortical osteogenic response to mechanical loading in female mice. (*A*) µCT analyses of cortical cross-sectional bone area of the mid-diaphyseal region of the non-loaded (Control) and loaded (Loaded) tibia in sham operated (Sham) and ovariectomized (Ovx) wild-type mice (*n* = 10). (*B*, *C*) Dynamic histomorphometric analyses of the cortical periosteal and endosteal surfaces (n = 4–5). In *B*, bone formation rate (BFR) data are presented as mean ± SEM; *p < 0.05 versus Control, Student's *t* test. In *C*, representative sections show similar loading-induced bone formation in Sham and Ovx mice both at the periosteal and endosteal surfaces (calcein/green and alizarin/red). The white bars represent 200 µm.

To determine the role of endogenous E2 in the cortical osteogenic response to loading, sham-operated (sham) and ovx WT mice were subjected to short periods of cyclic compressive loading of the right tibia, three times a week for 2 weeks while the left tibia was used as non-loaded control. µCT analyses of the mid-diaphyseal region of the tibia demonstrated that loading increased the cortical bone area by 26% (*p* < 0.01) compared with the control tibia in sham mice (Fig. 1*A*). Similar results were seen when the tibiae were analyzed by pQCT, demonstrating that the increased cortical bone area resulted in augmented cortical bone mineral content, polar moment of inertia and polar moment of resistance (see sham group Supplemental Table S1). To evaluate the effects of loading on the periosteal and endosteal surfaces, dynamic histomorphometric analyses were performed. The results demonstrate that the increased cortical bone area was mainly the result of a pronounced increased periosteal BFR and a slightly increased endosteal BFR (Fig. 1*B*, *C*). In sham mice, 81% of the loading-induced increase in cortical area was due to periosteal expansion and the remainder was due to endosteal new bone formation. The effect of loading on the periosteal BFR was reflected by a combination of increased mineralizing surface and mineral apposition rate (Supplemental Fig. S2). Importantly, the cortical osteogenic response to loading was unaffected in ovx mice compared with sham mice (Fig. 1 and Supplemental Fig. S2).

### ERα is required for the cortical osteogenic response to mechanical loading in female mice

**Figure 2:**
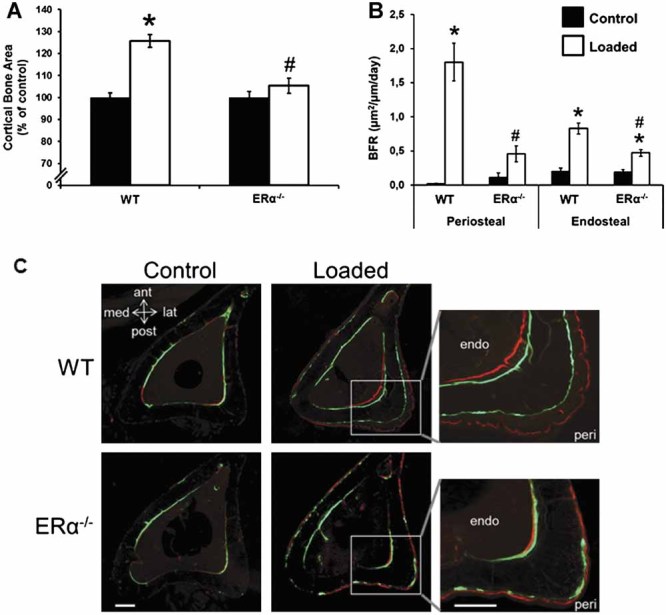
ERα is required for the cortical osteogenic response to mechanical loading in female mice. (*A*) µCT analyses of cortical cross-sectional bone area of the mid-diaphyseal region of the non-loaded (Control) and loaded (Loaded) tibia in wild-type (WT) and estrogen receptor-α inactivated (ERα^−/−^) mice (*n* = 8–10). (*B*, *C*) Dynamic histomorphometric analyses of the cortical periosteal and endosteal surfaces (*n* = 6–8). In *B*, bone formation rate (BFR) data are presented as mean ± SEM. The WT group in this figure is the same as the one described as Sham in Fig. 1; *p < 0.05 versus Control; #*p* < 0.05 effect of loading in ERα^−/−^ versus effect of loading in WT mice, Student's *t* test. In *C*, representative sections show that the loading-induced bone formation was severely reduced at the periosteal and slightly reduced at the endosteal surface in ERα^−/−^ compared with WT mice (calcein/green and alizarin/red). The white bars represent 200 µm.

We next evaluated the role of ERα in the cortical osteogenic response to loading using mice with a complete ERα inactivation.^26^ ERα^−/−^ mice displayed a severely reduced osteogenic response to loading with significantly smaller changes in cortical area (−78% ± 15%, *p* < 0.01) and BFRs at both the periosteal (−81% ± 9%, *p* < 0.01), and endosteal (−55% ± 12%, *p* < 0.05,) surfaces compared with the loading response in WT mice (Fig. 2). Changes in both mineralizing surface and mineral apposition rate were reduced at the periosteum in ERα^−/−^ compared with WT mice. Mineral apposition rate was also reduced endosteally in ERα^−/−^ mice (Supplemental Fig. S3). pQCT analyses further demonstrate that the cortical osteogenic response was impaired in ERα^−/−^ mice, with load-related changes in several cortical bone parameters being severely reduced, including cortical bone mineral content, polar moment of inertia, and polar moment of resistance (Supplemental Table S2). The loading-related increase in cortical bone area was also significantly reduced in ovx ERα^−/−^ mice compared with ovx WT mice (−55% ± 8%, *p* < 0.01). These findings demonstrate that ERα is required for a normal cortical osteogenic response in both the presence and absence of endogenous E2 (ligand).

### ERα AF-1 but not AF-2 is required for the cortical osteogenic response to mechanical loading in female mice

**Figure 3:**
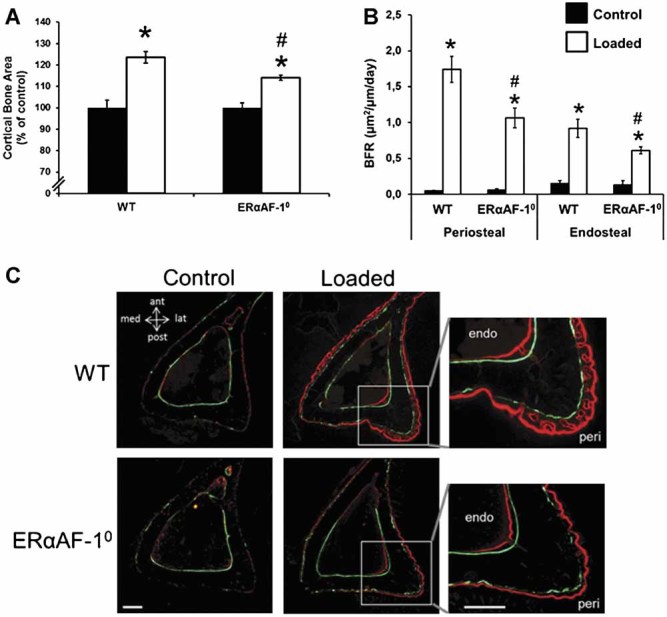
ERαAF-1 is required for the cortical osteogenic response to mechanical loading in female mice. (*A*) µCT analyses of cortical cross-sectional bone area of the mid-diaphyseal region of the non-loaded (Control) and loaded (Loaded) tibia in wild-type mice (WT) and in mice with specific inactivation of the estrogen receptor-α AF-1 (ERαAF-1^0^, *n* = 9–10). (*B*, *C*) Dynamic histomorphometric analyses of the cortical periosteal and endosteal surfaces (*n* = 9). In *B*, bone formation rate (BFR) data are presented as mean ± SEM; * *p* < 0.05 versus Control; # *p* < 0.05 effect of loading in ERαAF-1^0^ versus effect of loading in WT mice, Student's *t* test. In *C*, representative sections show that the loading-induced bone formation was reduced both at the periosteal and endosteal surfaces in ERαAF-1^0^ compared with WT mice (calcein/green and alizarin/red). The white bar represents 200 µm.

**Figure 4:**
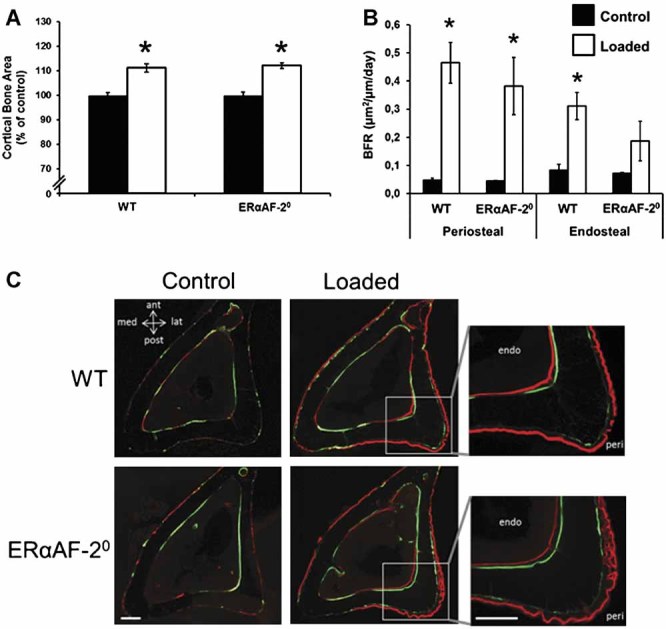
ERαAF-2 is not required for the cortical periosteal osteogenic response to mechanical loading in female mice. (*A*) µCT analyses of cortical cross-sectional bone area of the mid-diaphyseal region of the non-loaded (Control) and loaded (Loaded) tibia in wild-type (WT) mice and in mice with specific inactivation of the estrogen receptor-α AF-2 (ERαAF-2^0^, *n* = 7). (*B*, *C*) Dynamic histomorphometric analyses of the cortical periosteal and endosteal surfaces (*n* = 7). In *B*, bone formation rate (BFR) data are presented as mean ± SEM; * *p* < 0.05 versus control, Student's *t* test. In *C*, representative sections show similar loading-induced periosteal bone formation in WT and ERαAF-2^0^ mice (calcein/green and alizarin/red). The white bar represents 200 µm.

To characterize which domains of ERα are involved in the cortical bone response to axial loading, mouse models with specific inactivation of either AF-1 or AF-2 were evaluated. ERαAF-1^0^ mice displayed reduced osteogenic response to loading with changes in cortical area (−40% ± 11%, *p* < 0.05), and BFRs at both the periosteal (−41% ± 8%, *p* < 0.01), and endosteal (−45% ± 8%, *p* < 0.01) surfaces compared with WT mice (Fig. 3). Increases in periosteal mineral apposition rate, but not mineralizing surface, were significantly reduced in ERαAF-1^0^ mice compared with WT mice (Supplemental Fig. S4). Changes in cortical bone mineral content, polar moment of inertia and polar moment of resistance were also significantly reduced in ERαAF-1^0^ mice compared with WT mice (Supplemental Table S3). In contrast, in ERαAF-2^0^ mice the cortical periosteal osteogenic response to loading was unaffected compared with WT mice (cortical bone area: +11 ± 21%, periosteal BFR −22% ± 22%, nonsignificant; Fig. 4, Supplemental Fig. S5, and Supplemental Table S4). These findings demonstrate that ERαAF-1 but not ERαAF-2 is required for a normal cortical periosteal osteogenic response to mechanical loading in female mice.

### Loading does not affect ERE activation in cortical bone

**Figure 5:**
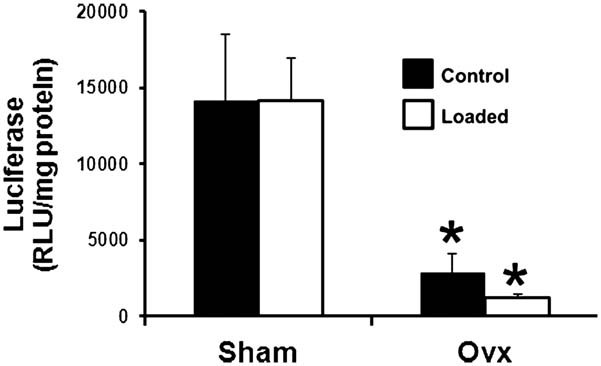
Effect of loading on ERE-mediated luciferase activity in cortical bone. Transgenic ERE-luciferase mice were loaded 8 hours before euthanasia. Luciferase activity per milligram of protein is given for the non-loaded (Control) and loaded (Loaded) tibial diaphyseal cortical bone in gonadal intact (Sham) and ovariectomized (Ovx) transgenic ERE-luciferase mice (*n* = 7). RLU = relative luciferase units. Data are presented as mean ± SEM. *p < 0.05 versus Sham, Student's *t* test.

To determine if the cortical osteogenic loading response involves activation of classical genomic ERE-mediated pathways, sham and ovx mice expressing a luciferase gene under control of an ERE-containing promoter were loaded for 3 or 8 hours before sacrifice. As expected, the ERE-activity was significantly higher in sham mice compared to ovx mice. However, loading did not affect luciferase expression significantly in cortical bone of sham or ovx mice (Fig. 5).

### The role of ERαAF-1 for the in vitro effect of strain on *Cox-2*, *Egr2*, and *IL-11* mRNA expression

**Figure 6:**
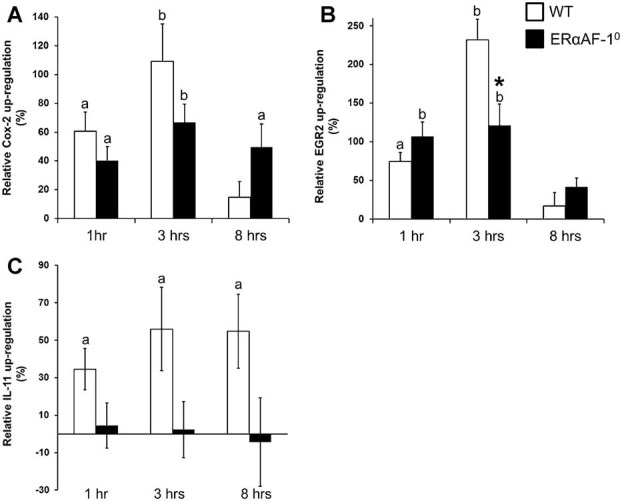
The role of ERαAF-1 for the effect of strain in vitro on *Cox-2*, *Egr2*, and *IL-11* mRNA expression. Passage 1 osteoblasts from wild-type (WT) mice and mice with specific inactivation of the estrogen receptor-α AF-1 (ERαAF-1^0^) were cultured on custom-made plastic slides and subjected to a single brief period of 600 cycles of four-point bending at a frequency of 1 Hz. The percentage upregulation of (*A*) *Cox-2*, (*B*) *Egr2*, and (*C*) *IL-11* mRNA levels at the indicated time points following strain is given. The percentage upregulation for this purpose is defined as: (value for each strained slide – mean static value)/mean static value * 100. Bars represent the mean upregulation ± SEM (*n* = 10–16 from 2–3 mice at each time point). ^a^*p* < 0.05, ^b^*p* < 0.01 for the upregulation at that time point; *p < 0.05 for the differences of effect of strain in ERαAF-1^0^ versus the effect of strain in WT at that time point.

COX2-mediated prostaglandin synthesis is known to activate a large number of rapidly diverging signaling pathways, which has recently been reported to be relevant to the regulation of *Sost* and *Ocn* by strain.^38^ WT and ERαAF-1^0^ cells subjected to strain significantly upregulated *Cox-2* mRNA expression after 1 hour relative to static controls (Fig. 6). *Cox-2* remained similarly upregulated at 3 hours after strain, but returned to levels not significantly different from static controls by 8 hours. The upregulation of *Cox-2* mRNA in ERαAF-1^0^ cells was not significantly different from that observed in WT at any time point, but *Cox-2* mRNA up-regulation remained significant 8 hours after strain in the ERαAF-1^0^ cells. There was a nonsignificant tendency of reduced strain induced upregulation of *Cox-2* mRNA in ERαAF-1^0^ cells compared with WT cells at the 3-hour time point (ERαAF-1^0^ cells showed 61% ± 12% of the upregulation observed in WT cells; Fig. 6*A*).

Of all the early strain target genes differentially regulated by loading, *Egr2/Krox-20* is associated with more pathways and functions than any other transcription factor.^39^ *Egr2* upregulation followed a similar time course as *Cox-2* in WT and ERαAF-1^0^ cells, with a significant upregulation observed 1 and 3 hours, but not 8 hours after strain in both cases (Fig. 6*B*). However, this response was significantly (*p* < 0.05) reduced in ERαAF-1^0^ cells compared with WT cells at the 3-hour time point (ERαAF-1^0^ cells showed 52% ± 12% of the upregulation observed in WT cells, Fig. 6*B*).

*IL-11* has been shown to be regulated by unloading and reloading in vivo^42^ and by fluid flow in vitro.^42–44^ *IL-11* was upregulated within 1 hour of strain and remained upregulated up to 8 hours later in WT cells, but not at any time point in ERαAF-1^0^ cells (Fig. 6*C*).

## Discussion

ERα is crucial for bones' adaptive response to loading but the relative roles of different ERα domains and the role of endogenous estrogens for this response are unclear. Using domain-specific ERα-inactivated mouse models subjected to a standardized axial tibia loading procedure, we herein demonstrate that AF-1 but not AF-2 in ERα is required for a normal cortical periosteal osteogenic response to mechanical loading and that endogenous E2 is dispensable for this response. In addition, we provide evidence that the loading response does not appear to involve activation of classical genomic ERE-mediated pathways in vivo.

Previous studies concerning the involvement of E2 in the osteogenic effect of loading have yielded conflicting results, and one may speculate that the divergent results are the consequence of differences in loading procedures (exercise, four-point bending, unloading, or axial loading), the bone evaluated (vertebra, ulna, or tibia), age (prepubertal or postpubertal) and gender.^4^, ^20–22^, ^24^, ^25^, ^45^ Furthermore, none of these studies included parallel evaluation of the ligand and ERα dependency for the osteogenic response to loading using the same loading procedure. In the present study, ligand and ERα dependency were tested using an identical axial loading procedure of the tibia in postpubertal female mice. Loading increased the cortical bone area as a result of a pronounced increase in periosteal bone formation and slightly increased endosteal bone formation. This osteogenic response was similar in intact and ovx WT mice. In contrast, ERα^−/−^ mice displayed a severely reduced cortical osteogenic response. These studies clearly demonstrate that ERα, but not endogenous E2, is required for the cortical osteogenic response to axial loading in the tibia of adult female mice.

Three previous studies have demonstrated that female K-ERα^−/−^ mice with compromised ERα expression display a reduced cortical osteogenic response to axial loading in the ulna.^12–14^ However, the K-ERα^−/−^ mouse model has a low expression of truncated ERα isoforms with unknown function, potentially affecting the results. In the present study, we used a complete ERα^−/−^ mouse model, and confirmed that ERα is indeed essential for the full osteogenic response to axial loading. These findings in ERα^−/−^ mice are consistent with the demonstration in vitro that osteoblast-like cells derived from ERα-depleted mice fail to proliferate in response to mechanical strain, and that this response can be restored by transfection of functional ERα.^13^

Previous in vitro studies suggest that ERα requires both AF-1 and AF-2 to mediate a proliferative response to strain.^37^ In addition, ligand-independent activation of ERα has been shown to occur via both AF-1 and AF-2.^46–50^ However, the in vivo roles of AF-1 and AF-2 in mediating the osteogenic response to loading were not possible to evaluate until the recent development of mouse models with separate and specific inactivation of either of these AFs.^1^, ^27^ In the present study, the loading response was evaluated using these ERαAF-1^0^ and ERαAF-2^0^ mouse models. Importantly, AF-1 but not AF-2 was required for a normal cortical loading response on the periosteal surface. Our findings provide strong evidence that ERα mediates the periosteal osteogenic response to loading by its AF-1 but not AF-2. A role of ERαAF-1 in the loading response is supported by in vitro findings demonstrating that strain phosphorylates Ser^122^ (mouse Ser^122^ = Human Ser^118^) within AF-1 in ERα and that phosphorylation of this serine in ERαAF-1 directs recruitment of promoter complexes and gene-specific transcription.^50^, ^51^

Although these experiments establish AF-1 to be the dominant transactivation domain in the ERα-mediated response of cortical bone to mechanical loading, other domains of ERα are probably also important for a normal loading response. This notion is supported by our observation that the loading response was more severely reduced in mice with complete ERα inactivation (ERα^−/−^ ≅ 80% reduction) than in mice with specific AF-1 inactivation in ERα (ERαAF-1^0^ ≅ 40% reduction).

We have recently demonstrated that the effect of E2 on cortical bone mass requires AF-2 but not AF-1 in ERα.^1^ The dissimilar roles of AF-1 and AF-2 for the loading response, requiring AF-1 but not AF-2, and E2 response, requiring AF-2 but not AF-1, in cortical bone, demonstrate that the signaling pathways for these ERα-mediated mechanisms differ. Separate ERα-mediated mechanisms are also supported by our finding that the cortical loading-response is E2 (ligand) independent.

The classical genomic mechanism of ERα action involves regulation of ERE-containing promoters. Earlier in vitro studies demonstrated that both strain and E2 increase the transcriptional activity from an ERE-reporter transiently transfected into an osteoblast cell-line, indicating that both strain and E2 enhance osteoblast activity via ERE-mediated mechanisms in vitro.^19^ To determine in vivo if the loading response involves activation of ERE-mediated pathways, the tibias of mice expressing a luciferase gene under the control of an ERE-containing promoter were loaded for 3 or 8 hours before euthanasia. We found that luciferase expression in cortical bone was not affected by loading, suggesting that the loading response does not require activation of classical genomic ERE-mediated pathways. A limitation with this substudy, exploring ERE-mediated pathways, was that only the 3-hour and 8-hour time points postloading were evaluated. However, we have in earlier experiments seen that E2-induced luciferase activity is maximal approximately 8 hours after treatment with E2.^52^

Although the present in vivo findings establish that the ERαAF-1 is important for the cortical osteogenic response to loading, there is no functional in vivo data evaluating the downstream mediators of this effect. Nevertheless, our recent in vitro data has demonstrated that the insulin-like growth factor (IGF)-I receptor physically associates with ERα in osteoblasts and we hypothesized that mechanical strain “primes” ERα via an unidentified mechanism (possibly involving its translocation to the membrane) to interact with the IGF-I receptor.^53^ Based on the present in vivo data, one might speculate that it is the AF-1 in ERα that interacts with the IGF-I receptor and that this interaction lowers the threshold levels of IGF-I necessary to stimulate IGF-I receptor activation, resulting in initiation of phosphatidylinositol 3-kinase/protein kinase B (AKT)-dependent activation of β-catenin and altered lymphoid-enhancing factor/T cell factor transcription, which in turn results in increased cortical bone formation.^53^

Activation of COX-2/PGE2 signaling is a robust response observed in numerous osteoblastic cell types subjected to various forms of mechanical stimulation. ERα has the potential to contribute to this pathway through various mechanisms, in the first instance by promoting *Cox-2* mRNA upregulation.^54^ There was a tendency of reduced upregulation of *Cox-2* mRNA in ERαAF-1^0^ osteoblasts compared with WT osteoblasts subjected to strain in vitro at the 3-hour time point but it did not reach statistical significance. The rather similar upregulation of *Cox-2* observed in WT and ERαAF-1^0^ osteoblasts subjected to strain in vitro might suggest that AF-1 functions of ERα are not required for this response in vitro. Alternatively, ERα's AF-1 mediated functions may contribute to this pathway downstream of COX-2. This is consistent with the finding that ligand-independent functions of ERα mediate ERK activation in osteoblastic cells subjected to strain.^41^ ERK activation downstream of PGE2 is involved in various strain responses including *Egr2* mRNA upregulation.^38^, ^39^ Contribution of ERαAF-1 to these responses is demonstrated by the blunting of *Egr2* upregulation in the ERαAF-1^0^ cells relative to WT controls. In addition to *Pge2*, *Egr2* expression is also regulated by IGF and Wnt signaling,^39^ both of which involve ERα.^53^, ^55^ That ERα normally facilitates a large number of interrelated pathways involved in the transcriptional regulation of *Egr2* is consistent with the finding that the transcriptomic response to loading is blunted and delayed in the bones of mice lacking ERα.^40^ IL-11 is involved in osteoblastic differentiation and has recently been shown to be mechanically regulated.^42^, ^56^ Whereas osteoblastic cells from WT mice upregulated *IL-11* at all time points tested following strain, cells from ERαAF-1^0^ mice did not show any significant changes in *IL-11* at any time point, suggesting that the AF-1 domain of ERα is required for this response. Mechanically-induced *IL-11* upregulation by fluid flow shear stress requires AP-1 sites, and mutations of these sites in the *IL-11* promoter prevent its upregulation.^42^, ^56^ Given that ERα is able to direct transcription through AP-1 sites,^57^ this suggests a mechanism whereby the loss of ERα AF-1 may directly impair regulation of gene expression. This is consistent with the demonstration that strain increases AP-1 reporter construct activity in WT osteoblastic cells, but not in similarly derived cells lacking ERα.^37^

In conclusion, ERα is required for the cortical periosteal osteogenic response to mechanical loading in a ligand-independent manner by its AF-1 but not AF-2. The dissimilar roles of AF-1 and AF-2 in the loading response in cortical bone (requiring AF-1 but not AF-2), and the E2 response (requiring AF-2 but not AF-1), demonstrate that the signaling pathways for these ERα-mediated mechanisms differ. In addition, we provide evidence which suggests that the cortical loading response does not involve activation of classical genomic ERE-mediated pathways. Further understanding of the ERα-mediated signaling pathways in the regulation of the cortical osteogenic response to loading might result in novel anabolic treatments targeting the cortical bone dimensions, which are the main determinants of bone strength.

## Disclosures

All authors state that they have no conflicts of interest.
