# Supplementary material for: Estrogen receptor-α is required for the osteogenic response to mechanical loading in a ligand-independent manner involving its activation function 1 but not 2
Source: J Bone Miner Res. 2013 Feb;28(2):291–301. doi: 10.1002/jbmr.1754 (PMC3575695; doi:10.1002/jbmr.1754)
Supplement: Supplementary file 6 [file jbmr0028-0291-sd6.doc]

**Supplemental Figure Legends**

**Figure S1 Expression and regulation of *SOST* and *DMP1* mRNA in long bone osteoblasts and bone.** (A) Long bone-derived osteoblasts do not express the osteocyte marker *SOST* but do express the late osteoblast/early osteocyte marker *DMP1*. No RT = negative control lacking reverse transcriptase. Bone = cortical bone (B) *DMP1* mRNA levels in long bone osteoblasts derived from wild type (WT) mice and mice with specific inactivation of the estrogen receptor-α AF-1 (ERαAF-1^0^) (C) The role of ERαAF-1 for the effect of strain *in vitro* on *DMP1* mRNA expression. Passage 1 osteoblasts from WT and ERαAF-1^0^ mice were cultured on custom-made plastic slides and subjected to a single brief period of 600 cycles of four-point bending at a frequency of 1 Hz. The percentage up-regulation of *DMP1* mRNA levels at the indicated time points following strain is given. The percentage up-regulation for this purpose is defined as: (value for each strained slide – mean static value) / mean static value * 100. Bars represent the mean up-regulation ± SEM, (n=10-16 from 2-3 mice at each time point).

**Figure S2 Endogenous estradiol is not required for the cortical osteogenic response to mechanical loading in female wild type mice.** Dynamic histomorphometric analyses of the cortical periosteal and endosteal surfaces of the non-loaded (Control) and loaded (Loaded) tibia in sham-operated (Sham) and ovariectomized (Ovx) wild type mice (n=4-5). (A) Mineralizing surface per bone surface (MS/BS). (B) Mineral apposition rate (MAR). Data are presented as mean ± SEM. * P < 0.05 vs. Control, Student`s t test.

**Figure S3 ERα is required for the cortical osteogenic response to mechanical loading in female mice**. Dynamic histomorphometric analyses of the cortical periosteal and endosteal surfaces of the non-loaded (Control) and loaded (Loaded) tibia in wild type (WT) and estrogen receptor-α inactivated (ERα^-/-^) mice (n=6-8). (A) Mineralizing surface per bone surface (MS/BS). (B) Mineral apposition rate (MAR). Data are presented as mean ± SEM. * P < 0.05 vs. Control. # P < 0.05 effect of loading in ERα^-/-^ vs. effect of loading in WT mice, Student`s t test.

**Figure S4 ERα AF-1 is required for the cortical osteogenic response to mechanical loading in female mice.** Dynamic histomorphometric analyses of the cortical periosteal and endosteal surfaces of the non-loaded (Control) and loaded (Loaded) tibia in wild type mice (WT) and in mice with specific inactivation of the estrogen receptor-α AF-1 (ERαAF-1^0^, n=9). (A) Mineralizing surface per bone surface (MS/BS). (B) Mineral apposition rate (MAR). Data are presented as mean ± SEM. * P < 0.05 vs. Control. # P < 0.05 effect of loading in ERαAF-1^0^ vs. effect of loading in WT mice, Student`s t test.

**Figure S5 ERα AF-2 is not required for the cortical periosteal osteogenic response to mechanical loading in female mice.**

Dynamic histomorphometric analyses of the cortical periosteal and endosteal surfaces of the non-loaded (Control) and loaded (Loaded) tibia in wild type (WT) mice and in mice with specific inactivation of the estrogen receptor-α AF-2 (ERαAF-2^0^, n=7) (A) Mineralizing surface per bone surface (MS/BS). (B) Mineral apposition rate (MAR). Data are presented as mean ± SEM. * P < 0.05 vs. Control, Student`s t test

**Supplemental Table Legends**

**Supplemental Table 1 Effect of loading on cortical bone parameters in female sham-operated (Sham) and ovariectomized (Ovx) wild type mice.** Cortical bone parameters were analyzed by pQCT in the mid-diaphyseal region of tibia. BMC = bone mineral content, MR = cortical cross sectional moment of resistance, MI = cortical cross sectional moment of inertia. Data are given for the loaded tibia in % over non-loaded tibia and presented as mean ± SEM (n=10). * P < 0.05 vs. non-loaded tibia, Student`s t test.

**Supplemental Table 2 Effect of loading on cortical bone parameters in female wild type (WT) and estrogen receptor-α inactivated (ERα^-/-^) mice.** Cortical bone parameters were analyzed by pQCT in the mid-diaphyseal region of tibia. BMC = bone mineral content, MR = cortical-cross sectional moment of resistance, MI = cortical-cross sectional moment of inertia. Data are given for the loaded tibia in % over non-loaded tibia and presented as mean ± SEM. ** The WT group in this table is the same as the one described as Sham in supplemental table 1. * P < 0.05 vs. non-loaded tibia (n=8-10). # P < 0.05 effect of loading in ERα^-/-^ vs. effect of loading in WT mice, Student`s t test.

**Supplemental Table 3 Effect of loading on cortical bone parameters in female wild type (WT) mice and in mice with specific inactivation of the estrogen receptor-α AF-1 (ERαAF-1^0^).** Cortical bone parameters were analyzed by pQCT in the mid-diaphyseal region of tibia. BMC = bone mineral content, MR = cortical-cross sectional moment of resistance, MI = cortical cross-sectional moment of inertia. Data are given for the loaded tibia in % over non-loaded tibia and presented as mean ± SEM. * P < 0.05 vs. non-loaded tibia (n=9-10). # P < 0.05 effect of loading in ERαAF-1^0^ vs. effect of loading in WT mice, Student`s t test.

**Supplemental Table 4 Effect of loading on cortical bone parameters in female wild type (WT) mice and in mice with specific inactivation of the estrogen receptor-α AF-2 (ERαAF-2^0^).** Cortical bone parameters were analyzed by pQCT in the mid-diaphyseal region of tibia. BMC = bone mineral content, MR = cortical-cross sectional moment of resistance, MI = cortical cross sectional moment of inertia. Data are given for the loaded tibia in % over non-loaded tibia and presented as mean ± SEM. * P < 0.05 vs. non-loaded tibia (n=7), Student`s t test.
