# Supplementary material for: Estrogen receptor-α is required for the osteogenic response to mechanical loading in a ligand-independent manner involving its activation function 1 but not 2
Source: J Bone Miner Res. 2013 Feb;28(2):291–301. doi: 10.1002/jbmr.1754 (PMC3575695; doi:10.1002/jbmr.1754)
Supplement: Supplementary file 7 [file jbmr0028-0291-sd7.doc]

**Table S1 Effect of loading on cortical bone parameters in female sham-operated (Sham) and ovariectomized (Ovx) wild type mice**

|  | **Sham** | **Ovx** |
| --- | --- | --- |
| BMC (% increase) | 29.4±2.4* | 33.3±3.3* |
| Bone area (% increase) | 24.6±2.4* | 25.8±3.0* |
| MR (% increase) | 26.4±2.7* | 24.6±3.7* |
| MI (% increase) | 36.1±3.5* | 31.3±3.6* |
