# Supplementary material for: Estrogen receptor-α is required for the osteogenic response to mechanical loading in a ligand-independent manner involving its activation function 1 but not 2
Source: J Bone Miner Res. 2013 Feb;28(2):291–301. doi: 10.1002/jbmr.1754 (PMC3575695; doi:10.1002/jbmr.1754)
Supplement: Supplementary file 8 [file jbmr0028-0291-sd8.doc]

**Table S2 Effect of loading on cortical bone parameters in female wild type (WT) and estrogen receptor-α inactivated (ERα^-/-^) mice**

|  | **WT**** | **ERα^-/-^** |
| --- | --- | --- |
| BMC (% increase) | 29.4±2.4* | 10.0±3.3***^#^** |
| Bone area (% increase) | 24.6±2.4* | 7.7±2.6***^#^** |
| MR (% increase) | 26.4±2.7* | 10.4±3.7***^#^** |
| MI (% increase) | 36.1±3.5* | 16.8±7.4***^#^** |
