# Supplementary material for: Estrogen receptor-α is required for the osteogenic response to mechanical loading in a ligand-independent manner involving its activation function 1 but not 2
Source: J Bone Miner Res. 2013 Feb;28(2):291–301. doi: 10.1002/jbmr.1754 (PMC3575695; doi:10.1002/jbmr.1754)
Supplement: Supplementary file 9 [file jbmr0028-0291-sd9.doc]

**Table S3 Effect of loading on cortical bone parameters in female wild type (WT) mice and in mice with specific inactivation of the estrogen receptor-α AF-1 (ERαAF-1^0^)**

|  | **WT** | **ERαAF-1^0^** |
| --- | --- | --- |
| BMC (% increase) | 29.4±3.0* | 19.3±2.5***^#^** |
| Bone area (% increase) | 23.8±2.8* | 16.3±1.9***^#^** |
| MR (% increase) | 31.7±4.3* | 20.8±2.2***^#^** |
| MI (% increase) | 32.9±3.8* | 22.5±2.3***^#^** |
