# Supplementary material for: Estrogen receptor-α is required for the osteogenic response to mechanical loading in a ligand-independent manner involving its activation function 1 but not 2
Source: J Bone Miner Res. 2013 Feb;28(2):291–301. doi: 10.1002/jbmr.1754 (PMC3575695; doi:10.1002/jbmr.1754)
Supplement: Supplementary file 10 [file jbmr0028-0291-sd10.doc]

**Table S4 Effect of loading on cortical bone parameters in female wild type (WT) mice and in mice with specific inactivation of the estrogen receptor-α AF-2 (ERαAF-2^0^)**

|  | **WT** | **ERαAF-2^0^** |
| --- | --- | --- |
| BMC (% increase) | 11.2±1.4* | 9.4±2.2* |
| Bone area (% increase) | 9.6±1.7* | 8.8±1.5* |
| MR (% increase) | 9.9±3.3* | 14.7±2.8* |
| MI (% increase) | 12.4±1.6* | 10.7±2.2* |
